# Surface Properties of Dental Materials Influence the In Vitro Multi-Species Biofilm Formation

**DOI:** 10.3390/polym18020288

**Published:** 2026-01-21

**Authors:** Sabina Noreen Wuersching, David Manghofer, Bogna Stawarczyk, Jan-Frederik Gueth, Maximilian Kollmuss

**Affiliations:** 1 Department of Conservative Dentistry, Periodontology and Digital Dentistry, LMU University Hospital, LMU Munich, Goethestrasse 70, 80336 Munich, Germany; d.manghofer@campus.lmu.de (D.M.); maximilian.kollmuss@med.uni-muenchen.de (M.K.); 2 Department of Prosthetic Dentistry, LMU University Hospital, LMU Munich, Goethestrasse 70, 80336 Munich, Germany; bogna.stawarczyk@med.uni-muenchen.de (B.S.); jan.gueth@med.uni-muenchen.de (J.-F.G.)

**Keywords:** 3D printing, fixed dental prostheses, bacterial adhesion, biofilm, surface properties

## Abstract

This study examined the association between biofilm growth and surface properties of 3D printed, milled, and conventional materials used for manufacturing fixed dental prostheses. Disc-shaped specimens were produced and finished from five 3D-printing resins (VarseoSmile Crown plus [VSC], NextDent C&B MFH [ND], VarseoSmile Temp [VST], Temp PRINT [TP], P Pro Crown & Bridge [P]), two polymer milling blocks (composite: TetricCAD [TC], PMMA: TelioCAD [TEL]), two conventional polymer materials (Tetric EvoCeram [TEC], Protemp 4 [PT]), and zirconia (ZR). Surface roughness (R_a_), wettability, interfacial tension (IFT) and surface topography were examined. Three-day biofilms were grown on the specimens using *A. naeslundii*, *S. gordonii*, *S. mutans*, *S. oralis*, and *S. sanguinis* in a multi-species suspension. Biofilms were quantified by crystal violet staining and with a plating and culture method (CFU/mL). Linear regression analysis was computed to demonstrate associations between the surface properties and biofilm growth. The strength of this relationship was quantified by calculating Spearman’s *ρ*. TC exhibited the highest, and TP the lowest IFT. TEC showed the highest R_a_, while TEL had the lowest, with significant differences detected particularly between milled and 3D-printed specimens. TP specimens exhibited the highest biofilm mass, while ZR surfaces retained the least. Bacterial viability within the biofilms remained similar across all tested materials. There was a strong negative correlation between total IFT and biofilm mass, and a moderate positive correlation between R_a_ and CFU/mL. Surface properties are shaped by material composition, microstructure, and manufacturing methods and play a crucial role in biofilm formation on dental restorations.

## 1. Introduction

Additive manufacturing technologies, commonly known as 3D printing, have attracted growing interest in recent years for processing dental materials, particularly polymers. Unlike CNC milling, where the restoration is cut from a solid block, 3D printing relies on a layer-by-layer construction approach that enables the creation of complex geometries. Among the available techniques, vat polymerization, particularly stereolithography (SLA) and digital light processing (DLP), has become the most commonly used method. These technologies are now widely implemented in dental laboratories and practices due to their ability to produce dental restorations suited to diverse clinical needs.

Today, 3D printing is routinely used for a variety of applications, such as the fabrication of dental models, surgical guides, and oral splints [1]. The development of photopolymerizable resins with good mechanical properties has enabled additive manufacturing of more complex geometries such as dental crowns, onlays, veneers, and bridges, which are generally referred to as fixed dental prostheses (FDPs). Over the past few years, several resin-based materials for the additive production of FDPs have been introduced. 3D-printed FDPs are emerging as promising alternatives for temporary dental restorations, particularly for treatments requiring long-term temporization, such as during dental implant therapy or full mouth reconstructions to increase the occlusal vertical dimension [2,3]. Furthermore, teeth with questionable endodontic or periodontal prognosis may require long-term temporization with FDPs, especially when they serve as abutment teeth to retain large prosthetic units [4]. The use of temporary FDPs may also be beneficial for visualizing the desired esthetic outcome during interventions involving alterations to the shape and size of anterior teeth [5].

For such FDPs to last in function for the required time, they must meet certain mechanical, physical and biological criteria. FDPs must provide a good marginal and internal fit to protect the underlying tooth structures, while emulating the individual tooth anatomy as naturally as possible to establish function and maintain the tooth position [6,7]. They must retain their integrity under functional loading by exhibiting high fracture resistance and low wear [8]. Furthermore, the colour stability of FDPs is critical to the long-term esthetics, especially in restorative procedures involving the anterior teeth [9]. As far as the biological properties are concerned, the two main requirements are good biocompatibility and low susceptibility to microbial adhesion [10]. Thick layers of plaque on the restoration surface can favour the development of secondary caries along the restoration margins or increase the risk of gum irritations and gingivitis [11,12]. Assuming that the biofilm matrix serves as a foundation for the incorporation of chromogenic pigments, the presence of plaque may also promote discolorations, thus affecting the esthetics of the restorations.

Inherent material properties such as the chemical composition and the surface properties have been shown to play a pivotal role in the bacterial adhesion on polymer-based restorative materials [13]. The most important variables defining the surface properties of a material are surface roughness, interfacial tension, and surface topography. However, there are inconsistent data about the association between surface properties and microbial adhesion on dental materials, with some studies finding a positive correlation between surface roughness and biofilm development, and others finding no such relationship [14,15]. One argument in favour of the idea that high surface roughness promotes microbial adherence is that a rough profile offers a larger area for biofilm formation and protects bacteria from shear forces during the initial attachment phase [16]. Several studies have postulated that surface topography, rather than surface roughness alone, plays the dominant role in preferred bacterial colonization [15]. While surface roughness describes only the fine, high-frequency irregularities of a material, surface topography captures the entire three-dimensional architecture of the surface, including its overall texture, waviness, peaks, valleys, and roughness features [17]. In addition, hydrophobicity and interfacial tension have been identified as further determinants of bacterial adhesion to polymer-based materials [18]. Despite growing interest in biofilm research, substantial gaps remain regarding the interactions between 3D-printed materials and complex biofilms. Since current investigations mainly rely on single-species models for assessing bacterial colonization on 3D-printed dental restorations, the effects of material surface characteristics on mature, multispecies oral biofilms are still poorly understood.

Since 3D printing is gaining popularity for the fabrication of FDPs, thorough examinations of the printable materials used are essential for assessing their clinical applicability. This study therefore addresses the research question of whether 3D-printed FDP materials differ from milled and conventionally fabricated FDPs in their surface properties and their susceptibility to bacterial biofilm formation. To answer this, we examined the surface properties of novel materials designed for 3D printing for both temporary and permanent FDPs and compare them with milled and conventionally produced counterparts. The null hypothesis is that there are no significant differences in surface properties or biofilm formation between 3D-printed, milled, and conventionally fabricated FDP materials.

## 2. Materials and Methods

### 2.1. FDP Materials

Ten different materials used for manufacturing FDPs were examined in this study: five printable resins, two polymer-based milling blocks (composite and PMMA) used for subtractive manufacturing, two types of materials with conventional curing processes (photo- and autopolymerization) and zirconium dioxide as the control group. Table 1 shows an overview of all materials along with the abbreviations used from this point on.

### 2.2. Manufacturing and Finishing of the Specimens

Disc-shaped specimens (diameter 10 mm, thickness 2 mm) were used for examining the surface properties and for studying biofilm growth. The disc template was designed using a CAD software (Tizian Creativ RT Version 3.2 Elefsina, Schütz Dental GmbH, Rosbach, Germany) and exported as a stl file. The template was sent to the designated manufacturing machine for each material. The DLP printer P30 (RapidShape GmbH, Heimsheim, Germany) was used for manufacturing VSC, VST, TP and P specimens. ND specimens were manufactured in the NextDent 5100 DLP printer (NextDent, Centurionbaan, The Netherlands). Washing and post-curing of the 3D printed specimens was performed strictly according to the manufacturers’ protocol. TC and TEL discs were produced by cutting 2 mm thick rectangular discs with a diamond saw, ensuring that each piece matched the surface area of the round discs. The two conventional resins were applied to a silicone mould forming the designated shape and were then either light-cured (TEC) in 2 mm layers with a dental LED polymerization light (intensity 1100 mW/cm^2^, exposure time 10 s, Bluephase Style, Ivoclar, Schaan, Liechtenstein) or allowed to self-cure (PT). ZR specimens were produced by milling the discs from a zirconia blank in a dental milling unit (CORiTEC 350i, imes-icore GmbH, Eiterfeld, Germany) and sintered according to the given protocol. The discs were finished on the day of fabrication in accordance with the finishing protocols recommended by the manufacturers. Detailed post-processing and finishing specifications for each material are shown in Table 2.

### 2.3. Surface Properties

#### 2.3.1. Wettability and Interfacial Tension

Wettability and interfacial tension (IFT) were examined using ten disc-shaped specimens of each material which were prepared and finished as described above. Specimens were rinsed under running distilled water to remove loose contaminants and then degreased by submerging them in 70% isopropyl alcohol for one minute. Specimens were placed in distilled water for another minute and then allowed to air dry before undergoing contact angle measurement. Contact angles of two different liquids differing in hydrophobicity (polar: deionized water; disperse: diiodomethane (CAS No. 75-11-6, Sigma-Aldrich, Steinheim, Germany)) were determined at room temperature (23 °C) with a contact angle device (EasyDrop, Krüss, Hamburg, Germany). For each test liquid, a drop with a defined volume (1 µL) was applied to the surface of each specimen and after 5 s the shape of the drop was captured by a camera. Each specimen was measured three times with both liquids. The contact angle of each drop (wettability) was evaluated using the DS software (DSA2.0, EasyDrop, Krüss, Hamburg, Germany). IFT [mN/m] was calculated using the method according to Owens, Wendt, Rabel and Kaelble (OWRK) [19].

#### 2.3.2. Surface Roughness Measurement

Surface roughness was assessed with a contact profilometer with a load of 0.00075 N (Profilometer M2, Mahr GmbH, Göttingen, Germany) using the same specimens as for the IFT measurement. Each specimen was measured three times in two different directions, with track length set to 4 mm and track spacing at 0.25 mm. The six measurements performed for each specimen were used to calculate the average surface roughness R_a_ (µm). The profilometer was calibrated with a reference block before the measurements were taken for each group.

#### 2.3.3. Observation of Surface Morphology via Scanning Electron Microscopy

The surface morphology of each specimen was observed in a scanning electron microscope (SEM). The specimens were attached to stubs and sputter-coated with a 10–20 nm thick Au-Pd layer for conductivity (SC762, Quorum Technologies, Laughton, UK). The specimens were examined under a field emission SEM (Zeiss Supra 55 VP, Carl Zeiss, Oberkochen, Germany) at an accelerating voltage of 10 kV and a working distance of 7–10 mm. Images were taken at 1K× and 10K× magnification. SEM imaging served solely descriptive purposes and was not intended for quantitative analysis.

### 2.4. Bacterial Biofilm Growth

#### 2.4.1. Bacterial Strains and Growth Media

The following bacterial strains were obtained from the German Collection of Microorganisms and Cell Cultures (DSMZ, Braunschweig, Germany): *Actinomyces naeslundii* (DSM 17233), *Streptococcus gordonii* (DSM 6777), *Streptococcus mutans* (DSM 20523), *Streptococcus oralis* (DSM 20627) and *Streptococcus sanguinis* (DSM 20567) were used. All strains were cultivated on Schaedler agar plates supplemented with Vitamin K1 and 5% Sheep Blood (Becton Dickinson, Franklin Lakes, NJ, USA). Brain-Heart-Infusion broth (BHI, Becton Dickinson) supplemented with Hemin (5 µg/mL) and Vitamin K1 (1 µg/mL) was used for culturing bacteria in liquid media. The bacteria were incubated at 37 °C with 60% humidity in an atmosphere containing 5.8% CO_2_.

#### 2.4.2. Biofilm Growth on Specimens

Biofilms were developed on disc-shaped specimens manufactured as described above. Cultures of *A. naeslundii*, *S. gordonii*, *S. mutans*, *S. oralis* and *S. sanguinis* were grown in BHI overnight to their individual stationary phase and subsequently diluted to an optical density corresponding to approximately 10^5^ CFU/mL [20]. Equal volumes of the five bacterial suspensions were combined and 2.5 mL of the mixture was dispensed into the wells of a 24-well plate. The discs were disinfected with 70% isopropyl alcohol for 1 min and left to air-dry. Subsequently, the specimens were placed into the wells containing the multi-species bacterial suspension and the well plate was incubated for 72 h (37 °C, 5.8% CO_2_), allowing the formation of mature biofilms on the disc surfaces. For further analyses of the grown biofilms, the specimens were rinsed with 0.9% NaCl solution to remove loosely attached cells.

#### 2.4.3. Quantification of the Total Biofilm Mass

The total biofilm mass was quantified with a crystal violet (CV) staining method by modifying a previously described protocol [21]. The specimens were immersed in a 0.1% aqueous crystal violet solution and incubated for 10 min at room temperature. The discs were rinsed in distilled water and excess staining solution was discharged by drying the discs with a clean paper towel. To solubilize the bound crystal violet, the discs were transferred to wells containing 30% acetic acid and incubated on an orbital shaker at 50 rpm for 10 min at room temperature. The resulting crystal violet solution/acetic acid solutions in each well were briefly mixed by pipetting, and duplicate aliquots from each specimen were transferred to an optically clear, flat-bottom 96-well plate. Optical density at 600 nm (OD_600_) was measured in a microplate reader (Varioskan Microplate Reader, Thermo Fisher Scientific, Waltham, MA, USA) and mean values of the two replicates were calculated for each disc.

#### 2.4.4. Number of Viable Bacteria Within the Biofilms

Biofilms grown on the surface of the discs were extracted using a modified three step method as previously described [20,22]. The discs were stored in reaction tubes containing 0.9% NaCl solution for one hour. The tubes were vortexed for 60 s, followed by 60 s sonication with an ultrasonic probe at 8 W, and further vortexing for 60 s. The tubes were cooled with ice during sonication to avoid thermal damage of the bacterial cells. Ten-fold serial dilutions of the sonicates were prepared in NaCl solution and plated on agar plates using a spread plate method. The agar plates were incubated for 48 h and the colony-forming units (CFUs) were counted following FDA guidelines (only plates with 25–250 colonies were considered). The number of viable cells extracted from the biofilms were calculated in terms of CFU/mL.

### 2.5. Statistical Analyses

All statistical analyses were implemented in Python 3.8.8 using the package *scipy*, *statmodels*, *scikit* and *pingouin* for inferential statistics and the packages *matplotlib* and *seaborn* for the descriptive analyses [23]. Homogeneity of variances was evaluated using Levene’s test, and data were assessed for normality with the Shapiro–Wilk test. Based on these assumptions, appropriate multiple group comparisons were applied: parametric and homoscedastic data were analyzed using ANOVA followed by Tukey’s HSD post-hoc test; non-parametric data were evaluated with the Kruskal–Wallis test and Dunn’s post hoc test with a non-negative false discovery rate correction; for heteroscedastic data that were predominantly normally distributed, Welch’s ANOVA and Games–Howell post-hoc test were used. A linear regression analysis was used to demonstrate possible associations between the surface properties and bacterial biofilm growth. The strength of the relationship was quantified by calculating Spearman’s rank correlation coefficient *ρ*. The alpha level was set to 0.05 for all statistical analyses.

## 3. Results

### 3.1. Surface Properties

#### 3.1.1. Wettability and Interfacial Tension

**Figure 1:**
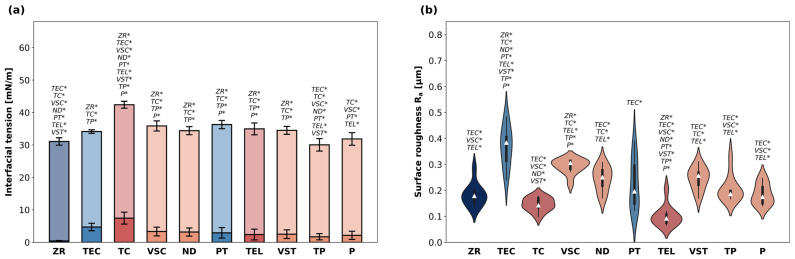
(**a**) Interfacial tension [mN/m] of the materials evaluated in this study. Data are presented as means and standard deviation of polar fractions (IFT_p_, saturated colours) and disperse fractions (IFT_d_, pale colours). *p*-values determined with ANOVA and Tukey’s HSD post hoc test using total IFT data (summary of IFT_d_ and IFT_p_). * indicates statistical significances with *p* < 0.05. (**b**) Surface roughness R_a_ of the specimens. Data shown as violin plots with the median (white triangle) and interquartile range. *p*-values determined with Welch’s ANOVA and Games–Howell post hoc test. * indicates statistical significances with *p* < 0.05.

The results for IFT are shown in Figure 1a. Contact angle values indicating surface wettability are displayed in Table 3. The average total IFT ranged between 30.0 and 42.4 mN/m. TC showed the highest IFT among all materials (*p* < 0.05 compared to all other materials except PT). The lowest IFT was registered for TP (*p* < 0.05 compared to all other materials except PT). There was a significant difference among the milled specimens, with TC showing a higher average total IFT than TEL (*p* < 0.05). Among the additively manufactured specimens, total IFT of VSC, ND and VST was in a similar range (34.4–35.6 mN/m), while TP and P exhibited a lower IFT. The largest polar fractions (IFT_p_) were registered for TC and TEC, whereas ZR showed the smallest IFT_p_. IFT_p_ of all other materials was in a similar range.

#### 3.1.2. Surface Roughness

Violin plots indicating surface roughness are visualized in Figure 1b. The average R_a_ values ranged between 0.10 and 0.37 µm. TEC showed the highest R_a_ (*p* < 0.05 compared to all other materials besides VSC) and TEL the lowest (*p* < 0.05 compared to all other materials besides TC). The second lowest R_a_ values were registered for ZR and TC specimens. Between the two materials with conventional curing processes, PT showed a lower surface roughness than TEC (*p* < 0.05). Furthermore, the violin plots provided an additional insight into the data distribution: most materials exhibited similar distribution patterns within their subgroups, which were characterized by a pronounced central region reflecting a high density of datapoints around the median. In contrast, TEC and PT data produced noticeably narrower violins with elongated tails, indicating a lower density of values near the center and revealing skewness in their distributions. VSC specimens exhibited the highest and P the lowest R_a_ among the five printable materials. The R_a_ values for all tested materials in descending order were TEC > VSC > VSZ > ND > PT > TP > P > ZR > TC > TEL.

#### 3.1.3. Surface Morphology

**Figure 2:**
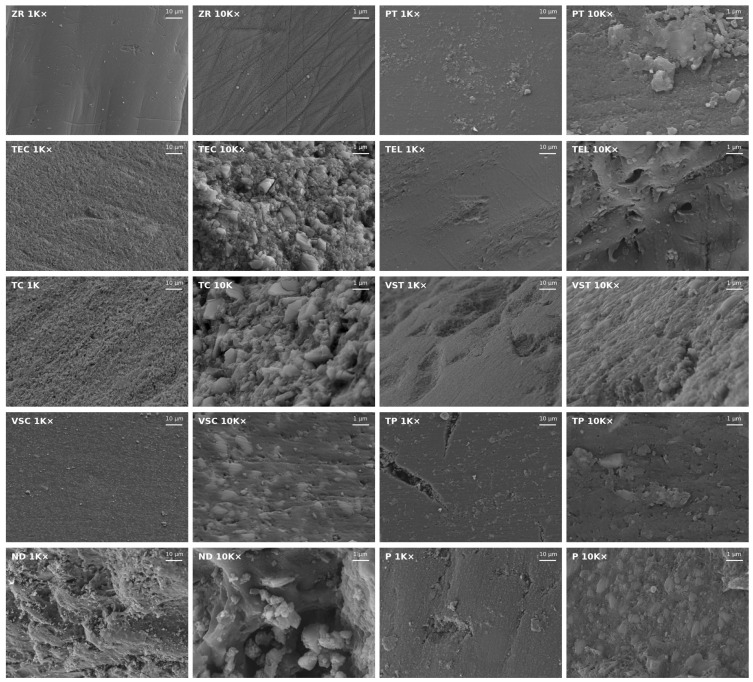
Representative SEM images of the surface of each material taken at 1K× and 10K× magnification.

Figure 2 shows SEM images of each material at 1K× and 10K× magnification. The ZR surface showed the least irregularities at 1K× magnification but exhibited straight fissures arranged in different directions at 10K× magnification. The surface texture of TEC and TC specimens was characterized by densely packed fillers in different sizes. Fillers were also visible on the surface of all 3D printed specimens, but they were overall fewer and less packed compared to TEC and TC. Moreover, the TP surface exhibited deep fissures and nanopores. The TEL surface seemed generally plane at 1K× magnification, but the 10K× magnification revealed surface irregularities such as randomly distributed micro-nano pores and grooves. The ND surface texture examined at 10K× magnification presented prominent waviness, accompanied by grooves containing filler conglomerates in different sizes.

### 3.2. Bacterial Biofilm Growth

**Figure 3:**
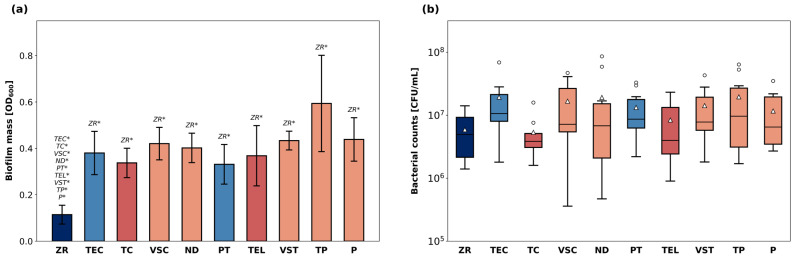
Bacterial biofilm growth on the surface of the discs. The total biofilm mass (**a**) after CV staining and solubilization is shown as OD_600nm_. *p*-values determined with Welch’s ANOVA and Games–Howell post-hoc test. * indicates statistical significances with *p* < 0.05. Number of viable cells (**b**) expressed as CFU/mL after plating and culturing biofilm extracts. Data are presented as medians (lines) and interquartile ranges (IQ) with whiskers extending to a maximum of 1.5 IQ. Triangles indicate means. Statistical testing was performed with Kruskal–Wallis test.

The total biofilm mass grown on the specimens is shown in Figure 3a. The highest average biofilm mass was detected on the surface of TP specimens. ZR surfaces led to the least biofilm retention and exhibited the lowest average OD600 among all materials (*p* < 0.05 compared to all other groups). Biofilm mass grown on all other materials was in a similar range.

The number of viable bacteria (CFU/mL) cultivated from biofilm sonicates is displayed in Figure 3b. The median bacterial counts ranged from 5.9 × 10^6^ CFU/mL (ZR) to 2.0 × 10^7^ CFU/mL (TP). There were no significant differences in terms of CFU/mL between the different materials.

### 3.3. Correlations Between Surface Properties and Bacterial Biofilm Growth

**Figure 4:**
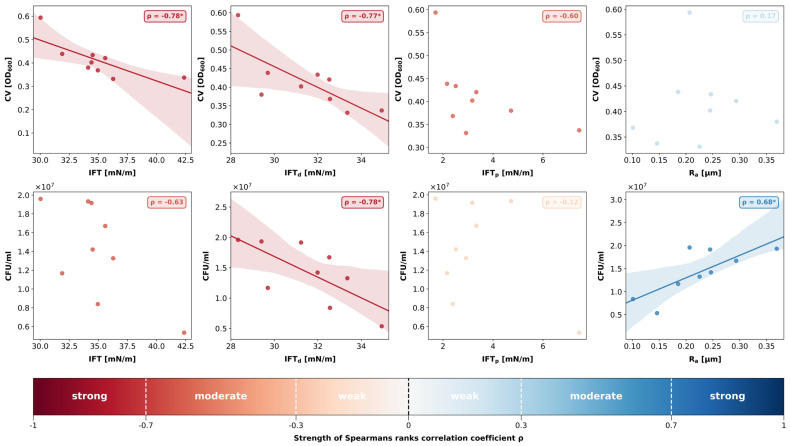
Scatterplots and linear regression (where applicable) demonstrating the relationship between the surface properties (mean IFT [mN/m], mean IFT_d_ [mN/m], mean IFT_p_ [mN/m], mean R_a_ [µm]) and bacterial biofilm growth (mean biofilm mass [OD_600nm_], median number of viable cells [CFU/mL]) for all polymer-based materials (ZR excluded from analysis). Strength of Spearman’s rank correlation coefficient *ρ* is visualized with a colour bar. * indicates statistical significances with *p* < 0.05.

Using the data obtained for the polymer-based materials, a linear regression analysis was computed to assess the relationship between the surface properties and bacterial biofilm growth. Next, the correlation strength was determined by calculating Spearman’s rank correlation coefficient *ρ* at an alpha level of 0.05. Both calculations were visualized in individual scatter plots using a colour scheme indicating the correlation strength (Figure 4). ZR was intentionally excluded from this analysis because its material class and surface chemistry differ markedly from polymers, potentially confounding the trends observed among the polymer-based materials. Limiting the regression to materials with comparable chemical and physical characteristics provided a more accurate assessment of how surface properties influence bacterial adhesion and biofilm development.

The correlation analysis revealed a strong negative correlation between total IFT and biofilm mass (*ρ* = −0.78; *p* = 0.01). While the disperse parts of IFT also showed a strong negative correlation with biofilm mass (*ρ* = −0.77; *p* = 0.02), the relationship between the polar parts of IFT and biofilm mass was not statistically significant (*ρ* = −0.60; *p* = 0.09). R_a_ and biofilm mass did not show any correlation (*ρ* = 0.17; *p* = 0.67).

A strong negative correlation was found between IFT_d_ and CFU/mL (*ρ* = −0.78; *p* = 0.01), whereas the polar parts of IFT did not correlate with the number of viable bacteria (*ρ* = −0.12; *p* = 0.77). Hence, the negative relationship between total IFT and CFU/mL was not statistically significant (*ρ* = −0.63; *p* = 0.07). However, a positive and statistically significant correlation coefficient was detected between surface roughness R_a_ and CFU/mL (*ρ* = 0.68; *p* = 0.04).

No significant correlation was found between the two microbiological parameters biofilm mass and CFU/mL (*ρ* = 0.5; *p* = 0.17).

## 4. Discussion

The surface properties of five commercially available resins used for additive manufacturing of FDPs and their effect on bacterial biofilm growth were examined and compared to milled and conventionally produced polymer-based materials. The materials used in this study were selected based on their designated clinical use. TEC is a nanohybrid composite used for direct tooth restorations and is characterized by its high filler content and the presence of prepolymers within its matrix. TEC has good mechanical properties in terms of wear, fracture resistance, colour stability and shows good long-term survival rates [24,25,26]. However, virtually all direct resin composites are limited by the fact that they are susceptible to marginal gap formation due to polymerization shrinkage and have to be light cured in layers to overcome this issue [27]. Therefore, resin composite milling blocks are becoming a popular alternative for polymer-based dental restorations. TC is a dental resin composite block used for CNC milling and has a similar composition as TEC but is industrially polymerized at high temperatures and under high pressure. This method, which is referred to as HT-HP polymerization, achieves a higher degree of conversion and leads to intrinsically more homogenous materials that come with fewer pores and irregularities compared to direct composites [10]. Despite the advantages of subtractive manufacturing, CNC milling has limitations that additive technologies can overcome. Three-dimensional printing enables the fabrication of complex geometries unrestricted by tooling size or milling angles and is more material-efficient and cost-effective, allowing multiple restorations to be produced simultaneously [28]. Composition-wise the printable resins are different from TEC and TC in that they have a higher monomer content and are less packed with fillers, which influences the mechanical properties and biocompatibility of the materials [10]. Further materials used for comparison were PT, a self-curing polymer-based material frequently employed for chairside fabrication of temporary restorations, and zirconia (ZR). Although not a polymer, ZR was considered an appropriate control group for comparison with the polymer-based material, as it is among the most widely used and durable options for permanent dental restorations [29].

The five-species biofilm model employed in this study was chosen to mimic a polymicrobial oral biofilm that is generally associated with frequent oral diseases. Since the placement of FDPs in the oral cavity is usually limited to the supragingival area, we opted for facultative anaerobic bacteria that are commonly found in biofilms associated with caries or gingivitis. A mixture of different streptococci was used because these species are pioneer colonizers during initial biofilm formation and enable the attachment of succeeding microorganisms [30]. *A. naeslundii* is frequently found in oral biofilms and, just like oral streptococci, is capable of withstanding a certain level of acidity in low-pH biofilms [31]. As oral biofilms progress from initial microbial adhesion to early structural organization, they typically begin transitioning into a more mature, multilayered network within two to three days. Based on this well-described developmental timeline, an incubation period of 72 h was considered appropriate for allowing sufficiently matured biofilms to form on the specimens. The methods used to assess bacterial biofilm growth focused on two complementary aspects: CV staining quantified the total biomass present on the specimens, including the extracellular biofilm matrix, whereas CFU/mL analysis of the biofilm extracts reflected the number of viable bacteria residing within the biofilm. The extraction of matured biofilms from the specimen surfaces was performed using a three-step protocol involving a combination of vortexing and sonication. This approach has previously been shown to provide the highest extraction efficiency compared with alternative methods [20].

As for surface roughness, the most notable differences were found between the different manufacturing techniques. While the R_a_ values of all 3D printed discs were in a similar range, the specimens milled from TC and TEL blocks exhibited lower R_a_ than the 3D-printed and the conventionally manufactured ones. This finding is consistent with recent data suggesting that milled FDPs tend to have smoother surfaces [32,33]. In addition to the HT-HP polymerization, which seems to have a positive effect on the surface roughness of TC and TEL, milled FDPs have the advantage that they are less affected by the operating conditions since there is no need for additional post-processing steps to obtain the final properties as with 3D printed restorations. The influence of operating conditions on surface roughness is highlighted by the markedly skewed Ra distributions of TEC and PT seen in the violin plots, which corresponds with the fact that they were the only specimens fabricated entirely by a human operator. Manual production likely introduced greater variability in surface quality compared with the more consistent surfaces of specimens fabricated by automated 3D printing and milling. Furthermore, it has been suggested that the material properties of 3D printed restorations may vary depending on the printing parameters, such as build orientation and layer thickness. While the printing orientation has been shown to influence the accuracy of dental restorations, with the best results typically achieved at a horizontal (0°) orientation, the surface quality seems to be less affected by the build angle [34]. In fact, recent studies indicate that printing orientation does not affect surface roughness at all, while layer thickness and printing technology seem to play a more significant role for the surface properties [35]. However, all specimens were polished following standard clinical protocols prior to further use, presumably levelling out the impact of build orientation on surface roughness [36].

In general, the R_a_ values of the 3D printed specimens were in an acceptable range and similar to recent data [37]. Nonetheless, ZR demonstrated the lowest surface roughness and appeared to have the smoothest surface topography in the SEM images, which is in line with findings reported in previous studies [38]. An R_a_ of 0.2 µm has been previously reported as threshold surface roughness, below which no further reduction in bacterial adhesion can be achieved [18,39]. ZR, TC, and TEL exhibited an average surface roughness that was lower than 0.2 µm, and, with the only exception of TEC, all other materials were also close to this value. The least amount of biofilm developed on ZR specimens, which agrees with previous data indicating that biofilms grown on the surface of ceramic materials are generally thinner than on resin-based surfaces [40,41]. Nonetheless, the bacteria within biofilms grown on ZR discs were highly viable, considering that the CFU/mL in the ZR group were not significantly lower than in the other material groups harbouring thicker biofilms. This inverse relationship between biofilm thickness and bacterial viability has been previously justified by limited nutrient supply in thicker biofilms, which therefore tend to contain less viable cells [41]. The correlation analysis indicated a positive correlation between R_a_ and CFU/mL but revealed no direct relationship between R_a_ and biofilm mass within the range of the R_a_ data of the polymer-based materials. This finding is generally consistent with previous data and supports the widely accepted notion that certain topographical characteristics, such surface depressions and irregularities, create favourable niches for bacterial cells, facilitating surface attachment and shielding the cells from shear forces during adhesion [42]. However, the apparent linearity between the CFU/mL and the surface properties must be interpreted with caution. While a monotonous trend is evident, the differences in CFU/mL remain biologically minor due to the exponential nature of bacterial growth. Moreover, the proportional relationship found between surface roughness and initial bacterial adhesion seems to level off when considering the entire biofilm along with its matrix, as no such relationship was found between R_a_ and total biofilm mass. As we relied mainly on R_a_ values to describe surface roughness, some aspects of surface complexity may not have been fully captured, and finer details of the texture might be underrepresented in our analysis. For example, R_a_ values of TP were in a similar range as the other 3D-printed specimens, but SEM images revealed fissures in the surface of the material that may have contributed to higher biofilm mass. Using R_z_ alongside R_a_ may help explain these unresolved relationships, since R_z_ captures extreme features, such as deep scratches or pits, more effectively. Overall, our statistical analyses reflect only the correlations observed in our in vitro data. To support any causal conclusions, these findings must be validated with larger datasets, as the small number of materials limits the strength of the current analysis.

Material chemistry and IFT are intricately connected because compositional and microstructural characteristics directly influence how the materials interact with different interfaces [18,43]. The two main components of polymer-based FDP materials are monomers and filler particles that come in different ratios depending on the designated manufacturing technique. While nanohybrid composites such as TEC and TC contain 70–80% wt fillers and only 20–30% wt methacrylates, the printable resins are, for reasons related to the manufacturing process, generally higher in monomer content and lower in filler particles [10]. Due to the polar nature of the mainly inorganic filler particles, materials with densely packed fillers at the surface, as seen in the SEM images of TEC and TC, exhibit larger polar fractions of IFT and lower water contact angles, indicating a high wettability. IFT_p_ of all other polymer-based materials was lower compared to TEC and TC, which concurs with the higher methacrylate content of 50–75%. Judged by the SEM images, these materials also exhibited a lower filler content compared to TEC and TC. Biofilm mass and IFT showed a strong negative relationship, implying that surfaces with lower IFT allow more total biofilm growth. Interestingly, both microbiological variables correlated strongly IFT_d_. While it is generally acknowledged that bacteria preferentially adhere to surfaces with high surface energy during initial surface attachment, our data demonstrate that mature biofilms seem to thrive on surfaces with lower IFT [14]. When judging the role of IFT in bacterial biofilm growth it is important to consider both the age and composition of the biofilm. Most studies demonstrating the relationship between IFT and microbial adhesion have focused on the early stages of biofilm formation, particularly within the first 24 h of development. However, relatively few studies have examined possible associations between surface properties and polymicrobial mature biofilms. Perhaps lower adhesion forces enable more effective matrix production and facilitate bacterial cohesion within the biofilm. In other words, biofilms grown on surfaces with lower IFT are less rigidly attached to the surface but appear to have a more recalcitrant architecture and greater cohesive strength. In fact, it is known that biofilm cohesiveness is largely influenced by extracellular polymeric substances (EPSs) such as polysaccharides, proteins, and nucleic acids, which fill the space between bacterial cells and form a protective gel-like matrix around cells. The composition and quantity of EPSs can vary greatly depending on the types of microorganisms, age of the biofilm and various environmental factors [44]. However, as different as the composition of the biofilm matrix may be, cohesive energy has been shown to increase with biofilm depth [45]. Put differently, the thicker the biofilms, the stronger the cohesion within the biofilm. Nonetheless, initial bacterial adhesion is a crucial step for enabling successive biofilm growth and depends on the surface affinity of early colonizers. Generally, microbiota found in the oral environment preferentially adhere to high free energy substrates which increases the attraction forces of pioneer colonizers [46]. Moreover, inherent characteristics of bacteria can impact early adhesion to surfaces and can vary greatly among the different species. For example, *S. mutans*, *S. oralis*, and *S. sanguinis* are considered hydrophobic bacteria that adhere readily to hydrophobic surfaces, whereas hydrophilic bacteria such as *S. mitis* are generally more attracted to hydrophilic surfaces [47]. Interestingly, *S. mutans* employed as mono-species culture has been shown to display a stronger propensity for adhesion on 3D printed surfaces than *S. sanguinis* but in which way these two species compete with one another during adhesion is not known [48].

In essence, early bacterial adhesion and biofilm thickness do not follow a linear pattern. Likewise, there is no consistent evidence in the literature supporting a direct correlation between mature biofilm mass and either surface roughness or IFT [46]. It should be mentioned that bacterial adhesion and successive biofilm formation is a complex process driven by numerous factors, many of which cannot be easily reproduced in an in vitro study. For example, factors related to the oral environment, such as pH, ionic strength of the medium, and the presence of shear forces, can affect biofilm thickness and composition. Moreover, van der Waals forces, electrostatic interactions and acid-base-bonding mediate bacterial adhesion but can also influence cohesiveness of the entire biofilm structure [40]. In addition to the surface properties examined in this study, surface charge plays a pivotal role in biofilm formation on dental restorations [49]. In vivo, the presence of the proteinaceous pellicle layer may alter the influence of wettability and IFT on biofilm formation. The absence of a salivary pellicle is also a significant limitation of our biofilm model. In vivo, the pellicle forms rapidly on oral surfaces and plays a key role in mediating initial bacteria adhesion. Introducing a pellicle layer before bacterial inoculation could have altered the pattern and extent of biofilm development, potentially leading to different adhesion dynamics and growth behaviour. Since no artificial ageing or functional loading was performed in this study, our findings represent surface properties at the initial manufacturing stage and do not account for long-term behaviour. Moreover, biofilm growth on polymer-based surfaces is known to cause surface deterioration, potentially enhancing long-term biofilm formation. Variations in material composition and exposure to environmental conditions such as moisture, temperature fluctuations, and mechanical stress may further impact surface degradation over time. Evaluating alternative post-processing procedures, instead of relying solely on manufacturers’ recommended protocols as we did, may optimize surface characteristics and potentially alter bacterial adhesion outcomes. Further studies incorporating these factors are necessary to confirm our results and provide a more comprehensive understanding of long-term performance.

## 5. Conclusions

Understanding how surface properties affect biofilm formation is key to preventing caries and gingivitis in patients with dental restorations. In general, in vitro biofilm growth on 3D-printed specimens was comparable to that observed on conventional polymer-based materials, both in terms of total biofilm mass and number of viable bacteria. Interfacial tension appeared to be strongly influenced by compositional and microstructural characteristics, particularly the inorganic fillers within the methacrylate matrix. Milled materials produced through industrial polymerization exhibited a lower surface roughness than 3D-printed and conventionally manufactured ones. Because biofilm age and composition can markedly affect adhesion behaviour, these factors must be considered when comparing findings across studies. The generally acceptable properties of 3D-printed materials support their potential as viable options for both temporary and permanent dental restorations.

## Figures and Tables

**Table 1:** Materials used in this study.

	Material	Abbr.	Manufacturer	Material Type (Manufacturing Technique)	LOT-No.
permanent FDP	Tizian Blank Translucent Zirconium Dioxide	ZR	Schütz Dental (Rosbach, Germany)	Zirconium dioxide (subtractive)	2019000511
	Tetric EvoCeram	TEC	Ivoclar (Schaan, Liechtenstein)	Nanohybrid composite (conventional, photopolymerizing)	Z00JJD
	Tetric CAD	TC	Ivoclar	PMMA milling block (subtractive)	Z0159M
	VarseoSmile Crown plus	VSC	BEGO (Bremen, Germany)	3D-printing resin (additive)	600324
	NextDent C&B MFH	ND	NextDent (Centurionbaan, The Netherlands)	3D-printing resin (additive)	XG181N20
temporary FDP	Protemp 4	PT	Solventum (Saint Paul, MN, USA)	Bis-acrylic composite (conventional, autopolymerizing)	7420901
	Telio CAD	TEL	Ivoclar	PMMA milling block (subtractive)	Z0179Y
	VarseoSmile Temp	VST	BEGO	3D-printing resin (additive)	600213
	Temp PRINT	TP	GC Europe (Leuven, Belgium)	3D-printing resin (additive)	2008071
	P Pro Crown & Bridge	P	Straumann (Basel, Switzerland)	3D-printing resin (additive)	210514A

**Table 2:** Post-processing steps of each material according to the manufacturer’s protocol.

Material	Post-Processing and Finishing Steps
ZR	3-step polishing with silicone polishing burs for zirconia (Turbo shine lab medium/fine/extra fine, Acurata, Thurmansbang, Germany)
TEC	Surface buffing with a rotating disc sander (P240 grit size), polishing with composite silicone polishing burs (Set 4669, Komet Dental/Brasseler, Lemgo, Germany)
TC	2-step polishing with composite silicone polishing burs (Set 4669, Komet Dental/Brasseler)
VSC	3 min ultrasonic precleaning with 96% Ethanol, 2 min ultrasonic cleaning with fresh 96% Ethanol, jet polishing (1.0 bar), 2 × 90 s polymerization (200 W), surface buffing with a rotating disc sander (P240 grit size), pre-polishing with water/pumice in a dental lathe machine (goat hair brush, 1500 rpm), high -gloss polishing with polishing paste (goat hair brush, 1500 rpm)
ND	3 min ultrasonic precleaning with 96% Ethanol, 2 min ultrasonic cleaning with fresh 96% Ethanol, 10 min UV post polymerization at 60° C, surface buffing with a rotating disc sander (P240 grit size), pre-polishing with water/pumice in a dental lathe machine (goat hair brush, 1500 rpm), high -gloss polishing with polishing paste (goat hair brush, 1500 rpm)
PT	Surface buffing with a rotating disc sander (P240 grit size), pre-polishing with water/pumice in a dental lathe machine (goat hair brush, 1500 rpm), high -gloss polishing with polishing paste (goat hair brush, 1500 rpm)
TEL	pre-polishing with water/pumice in a dental lathe machine (goat hair brush, 1500 rpm), high -gloss polishing with polishing paste (goat hair brush, 1500 rpm)
VST	3 min ultrasonic precleaning with 96% Ethanol, 2 min ultrasonic cleaning with fresh 96% Ethanol, jet polishing (1.0 bar), 2 × 90 s polymerization (200 W), surface buffing with a rotating disc sander (P240 grit size), pre-polishing with water/pumice in a dental lathe machine (goat hair brush, 1500 rpm), high -gloss polishing with polishing paste (goat hair brush, 1500 rpm)
TP	2 min ultrasonic precleaning with 96% Ethanol, 2 min ultrasonic cleaning with fresh 96% Ethanol, 10 min UV post polymerization, surface buffing with a rotating disc sander (P240 grit size), pre-polishing with water/pumice in a dental lathe machine (goat hair brush, 1500 rpm), high -gloss polishing with polishing paste (goat hair brush, 1500 rpm)
P	2 min ultrasonic precleaning with 96% Ethanol, 2 min ultrasonic cleaning with fresh 96% Ethanol, jet polishing (1.0 bar), 10 min UV post polymerization, surface buffing with a rotating disc sander (P240 grit size), pre-polishing with water/pumice in a dental lathe machine (goat hair brush, 1500 rpm), high -gloss polishing with polishing paste (goat hair brush, 1500 rpm)

**Table 3:** Contact angles indicating the surface wettability of the materials in the presence of polar (water) and apolar (diiodomethane) liquids.

Material	θ_water_	θ_diiodomethane_
ZR	99.37 ± 1.30	56.37 ± 1.98
TEC	83.20 ± 3.20	58.55 ± 0.94
TC	73.14 ± 3.75	48.82 ± 1.93
VSC	86.95 ± 6.21	54.57 ± 3.18
ND	86.66 ± 4.48	55.35 ± 2.17
PT	86.25 ± 4.90	51.62 ± 2.29
TEL	88.89 ± 5.85	53.03 ± 3.24
VST	88.50 ± 5.28	54.02 ± 2.16
TP	95.77 ± 5.28	60.27 ± 3.09
P	89.61 ± 5.74	57.60 ± 3.10

## Data Availability

The original contributions presented in this study are included in the article. Further inquiries can be directed to the corresponding author.
